# Inactivated rotavirus vaccine by parenteral administration induces mucosal immunity in mice

**DOI:** 10.1038/s41598-017-18973-9

**Published:** 2018-01-12

**Authors:** Theresa K. Resch, Yuhuan Wang, Sung-Sil Moon, Jessica Joyce, Song Li, Mark Prausnitz, Baoming Jiang

**Affiliations:** 1 Division of Viral Diseases, Centers for Disease Control and Prevention (CDC), Atlanta, Georgia USA; 2 School of Chemical & Biomolecular Engineering, Georgia Institute of Technology, Atlanta, Georgia USA

## Abstract

To improve the safety and efficacy of oral rotavirus vaccines, we developed an inactivated rotavirus vaccine (IRV) for parenteral administration. Since it remains unknown whether parenteral vaccination can induce mucosal immunity, we performed a comprehensive assessment of immune responses to IRV in mice with an adjuvant-free dissolving polymer MN patch or by alum-adjuvanted IM injection. We demonstrated that IRV induced the expression of the gut homing receptor LPAM-1 on T and B cells in spleen and mLN of vaccinated mice. MN patch IRV vaccination induced a slight Th1 phenotype while IM vaccination induced a balanced Th1/Th2 phenotype. In addition, a dose-sparing effect was seen for rotavirus-specific serum IgG and neutralizing activity for both vaccination routes. Our study is the first to show that parenterally administered IRV can induce mucosal immunity in the gut, in addition to strong serum antibody response, and is a promising candidate vaccine in achieving global immunization against rotavirus.

## Introduction

Despite the introduction of two live oral vaccines (Rotarix and RotaTeq^TM^) in many countries, rotavirus (RV) is still the leading cause of severe gastroenteritis and diarrhea, resulting in estimated 215,000 deaths among children under the age of 5 worldwide per year^1^. The two vaccines have shown high efficacy in high and middle-income countries. However, reduced vaccine-induced protection in low income countries, large cold chain storage, the low risk of intussusception, and vaccine-associated diarrhea are lingering concerns.

Since oral vaccination is subject to risk factors, such as rich microbiome and enteropathy in the gut, and is often associated with reduced immunogenicity, most vaccines are delivered by parenteral intramuscular (IM) vaccination in infants and adults. IM administration has shown to be safe, efficient in inducing an immune response and generally accepted by the population. Likewise, an IM administered inactivated rotavirus vaccine (IRV) would be less prone to risk factors associated with oral vaccination including intussusception and diarrhea induction in vaccinated infants and potentially be more immunogenic and efficacious among children in low-income countries^2^.

In recent years, skin vaccination using dissolving microneedle (MN) patches has been pursued as a potential alternative to IM administration. MN patches may not need a cold chain, can be applied by minimally trained personnel, and do not create sharp biohazardous waste^3^. In addition, large-scale MN patch fabrication could be cost-effective and has been proven to be thermostable for dried vaccines^4–8^. The delivery of vaccines to the skin by MN patch has been studied for a number of vaccines, including rabies, influenza, hepatitis B, and polio^9–13^. The vaccine is directly delivered to antigen presenting cells (APC) in the epidermis, and thus may result in dose-sparing effects and better vaccine-induced protection^14–16^.

IRV CDC-9 administered by IM injection or by skin vaccination using a MN patch or device was effective in inducing IgG, IgA, homotypic and heterotypic neutralizing antibody in serum and strong protection against infection and diarrhea from oral challenge with a virulent human RV in pre-clinical studies^2,17–19^. However, it remains unknown whether parenteral vaccination can induce mucosal immunity in the intestine of animals and humans. Natural rotavirus infection or oral vaccination induces systemic immunity and strong serum antibody response in children. However, the mechanism and role of mucosal immunity in RV infection and immunity is not fully understood. Generally, lymphocytes expressing the gut-homing receptor LPAM-1 home with a higher frequency to Payer’s patches and mesenteric lymph nodes than LPAM-1 negative cells^20^. In children with RV infection, a very small number of circulating T and B cells are activated to express LPAM-1 and the LPAM-1^+^ B cells were shown to secrete RV-specific antibody^21–23^. However, there are no defined correlates of protection for LPAM-1 expression after natural RV infection or oral RV vaccination. No studies have investigated the role of B and T cells expressing LPAM-1 after parenteral RV vaccination.

In this study, we examined systemic and mucosal immune responses to IRV vaccination in mice using an adjuvant-free, dissolving MN patch or alum-adjuvanted IM injection. We showed that IM and MN patch vaccination triggered the activation of memory B and effector memory T helper cells expressing LPAM-1 primarily in spleen and mLN. In addition, RV-specific IgG antibodies and neutralizing activities in serum were expressed in a dose-dependent manner. The results of our study provide additional evidence for furthering development of CDC-9 IRV for parenteral vaccination against RV among children throughout the world.

## Results

### Determination of antigen dose administered by microneedle patches

**Figure 1:**
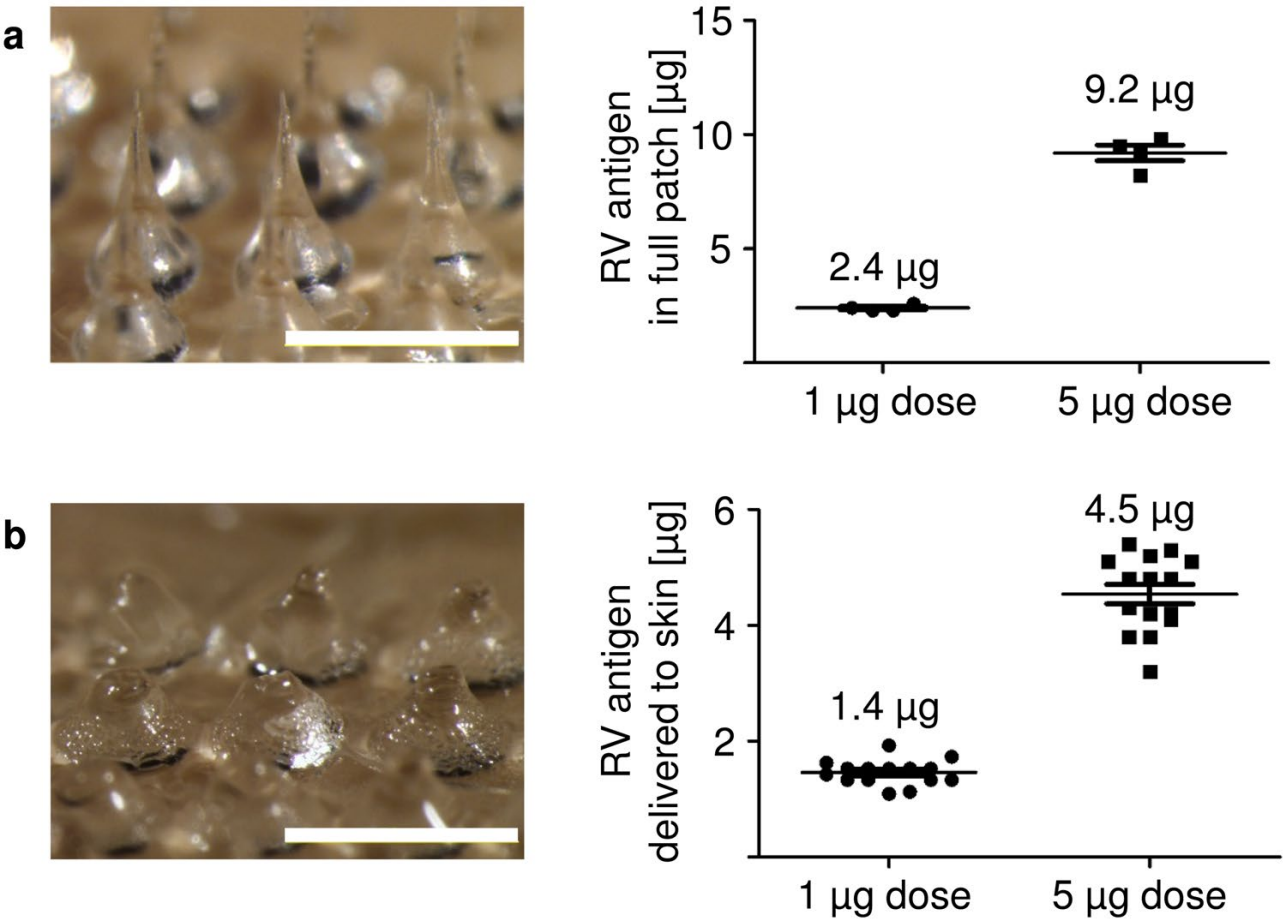
Delivery efficiency of IRV by microneedle patch. (**a**) Left: One representative microneedle (MN) patch before insertion into the skin of mice. Intact patches contain 100 MN in a pattern of 10 × 10 (scale bar: 1 mm). Right: Four MN patches were analyzed before every vaccination to determine the antigen amount coated to the needles. MN patches were produced with 2 µg (to deliver 1 µg) and 10 µg (to deliver 5 µg) inactivated rotavirus antigen. (**b**) Left: One representative MN patch after insertion into the skin of mice showing dissolution of the MN tips (scale bar: 1 mm). Patches were collected after insertion for microscopic analysis. Right: Calculation of the delivered antigen by measuring the residual rotavirus antigen amount on the patches after insertion. For (**a**) n = 4 and (**b**) n = 15, mean and standard deviation (s.d.). Shown are representative pictures and data from dose 2 vaccination. Analysis was performed before and after all three vaccinations. MN: Microneedle; RV: Rotavirus; IRV: Inactivated rotavirus vaccine.

MN patches used for RV vaccination were fabricated as 100 MN arrays, containing RV antigen in the MN tips. Imaging analysis showed sharply tapered MN tips on widely tapered base structures before use (Fig. 1a, left). Based on prior experience, approximately 50% of the antigen loaded in the patches is contained in the MN tip portion that penetrates the skin. Therefore, aimed antigen content in each patch was 2 µg (to deliver 1 µg antigen) and 10 µg (to deliver 5 µg antigen). Measurement of RV antigen showed that on average, patches contained 2.4 µg and 9.2 µg RV antigen (Fig. 1a, right). After insertion into the skin of mice, the inserted tips of MN patch dissolved away, and the wide base structures that were not inserted into the skin remained (Fig. 1b, left). Measurement of residual RV antigen in the used patches showed that the average dose delivered to the skin was 1.4 µg and 4.5 µg, aiming for 1 µg and 5 µg doses, respectively (Fig. 1b, right).

### IRV vaccination leads to a shift of Th and Tc cells in peripheral blood

To determine whether IRV vaccination led to any changes in the immune cell distribution, mice were inoculated with IRV either by IM injection or by MN patches on day 0, 21, and 42 and organs before vaccination (baseline), two days after each vaccination (day 2, 23, and 44), and at study end (day 63) were harvested and examined to assess systemic (blood and spleen) and intestinal (mLN) immune cell distribution by flow cytometry (Supplementary Figs 2 and 3). Approximately 40% of total cells in blood, spleen, and mLN of unvaccinated animals (baseline) are CD3-expressing T cells (38% in blood, 37% in spleen, and 42% in mLN). A more detailed analysis of T cells showed 59–63% T helper (Th) cells co-expressing CD3 and CD4, and 37–40% cytotoxic T (Tc) cells co-expressing CD3 and CD8 in blood, spleen and mLN, respectively. B cells expressing B220 were present in blood (33%), spleen (39%) and mLN (24%). We also analyzed organs for the presence of granulocytes (CD11b^+^GR-1^+^), NK cells (NK1.1^+^), dendritic cells (CD11b^+^CD11c^+^), and myeloid cells (CD11b^+^F4/80^+^) and detected no elevations. We observed no differences in the distribution of T cells, B cells, dendritic cells, myeloid cells, and NK cells in blood, spleen and mLN of animals that received different IRV doses (1 µg, 5 µg) and placebo via IM or MN patch routes when compared to baseline values (Supplementary Fig. 3). However, we observed that both vaccination routes independent of the vaccination dose induced a comparable shift in Th/Tc cell ratio towards a Th cell phenotype in blood on days 23, 44 and 63 post vaccination. No shift in Th/Tc ratio was seen in spleen and mLN of vaccinated mice.

### IRV vaccination induces B and T cells expressing the gut-homing receptor LPAM-1

**Figure 2:**
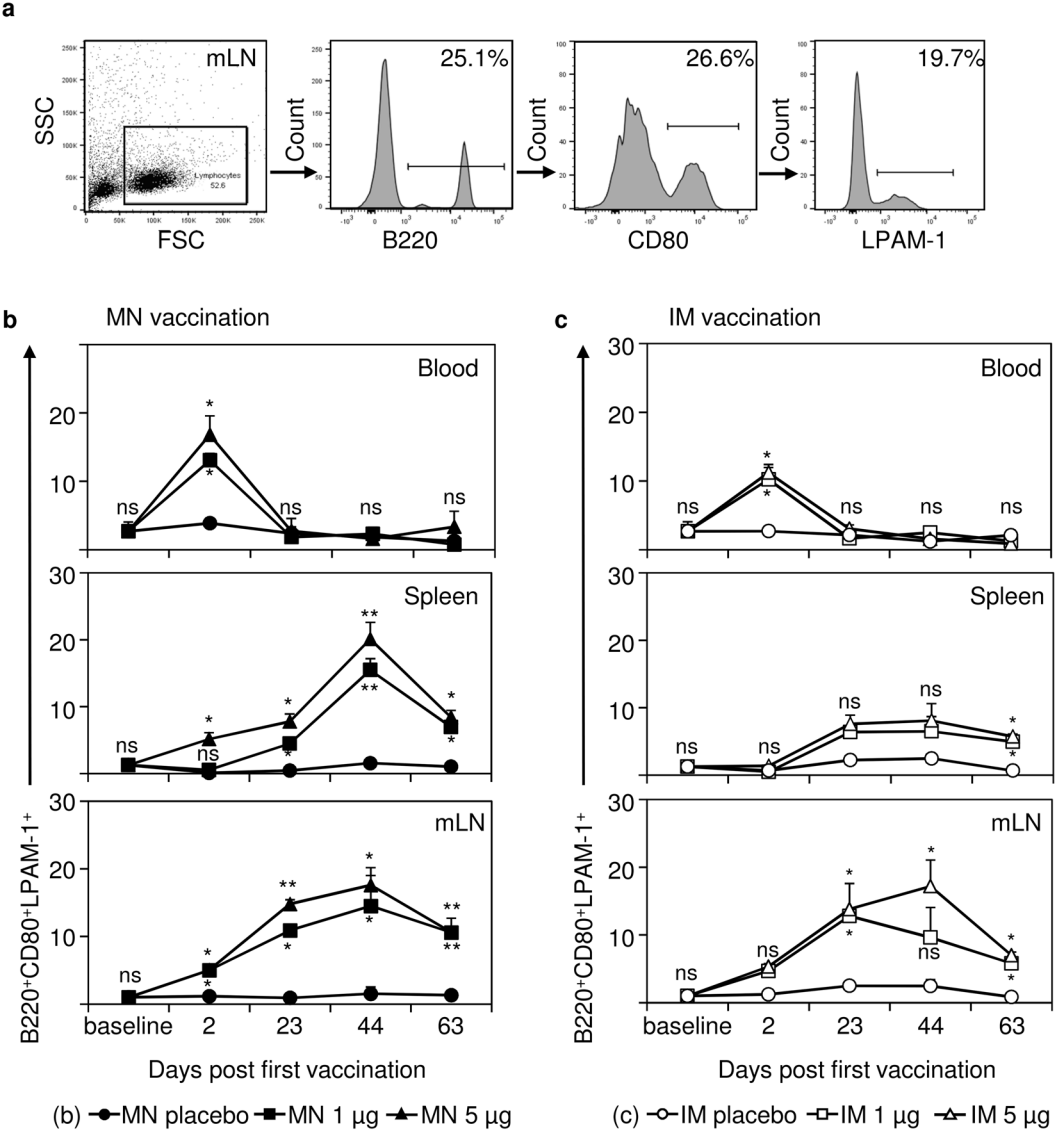
Vaccination with IRV leads to the expression of the gut homing receptor LPAM-1 on B cells in blood, spleen and mesenteric lymph node (mLN). Mice were vaccinated with IRV either via the microneedle (MN) patch or intramuscular (IM) injection with the indicated doses or with a placebo control. Organs and peripheral blood were collected from unvaccinated animals (baseline), 2 days after each dose (day 2, 23, and 44) and at the study end (day 63). (**a**) Percentages of surface marker positive cells were determined by gating on the positive population in the appropriate histogram. (**b**) Proportions of B220^+^CD80^+^LPAM-1^+^ (activated B cells) in blood, spleen, and mLN at the indicated time points after MN patch vaccination. (**c**) Proportions of B220^+^CD80^+^LPAM-1^+^ in blood, spleen, and mLN at the indicated time points after IM vaccination. Data shown in (**a**) are representative of one mouse vaccinated with 5 µg IRV on day 44. Shown in (**b**) and (**c**) are the mean and s.d. of data from 4 (baseline) or 5 mice. Mann-Whitney test was used for statistical analysis comparing vaccinated animals with appropriate placebo controls. Upper p value indicator for 5 µg IRV vaccination; lower p value indicator for 1 µg IRV vaccination. If only one p value indicator is present, this indicator applies to both, 1 µg and 5 µg IRV vaccination. ns: not significant p > 0.05; *p ≤ 0.05; **p ≤ 0.01; MN: microneedle; IM: intramuscular; mLN: mesenteric lymph node.

**Figure 3:**
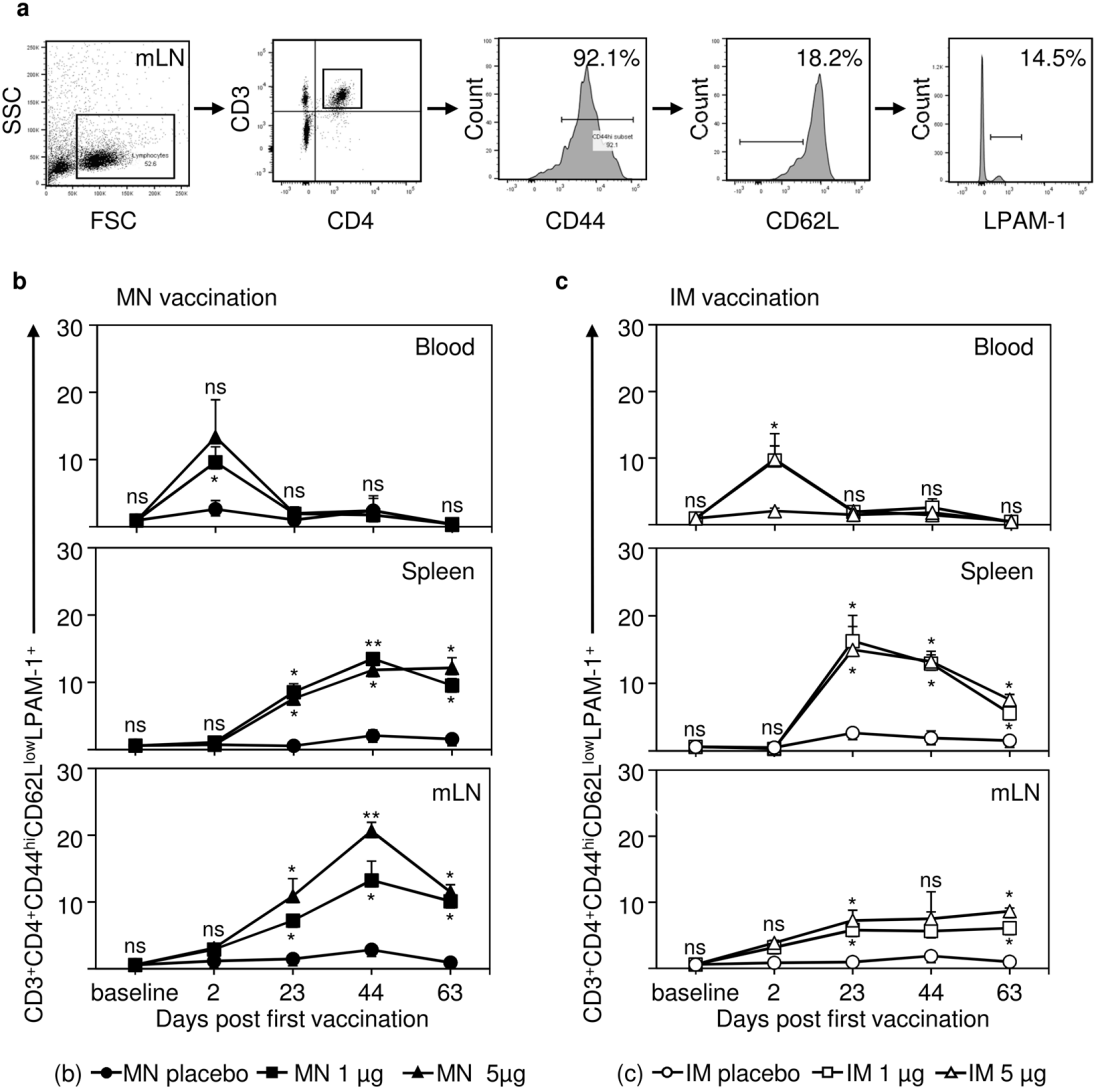
Vaccination with IRV leads to the expression of the gut homing receptor LPAM-1 on Th cells in blood, spleen and mesenteric lymph node (mLN). Mice were vaccinated with IRV either via the microneedle (MN) patch or intramuscular (IM) injection with the indicated doses or with a placebo control. Organs and peripheral blood were collected from unvaccinated animals (baseline), 2 days after each dose (day 2, 23, and 44) and at the study end (day 63). (**a**) Percentages of surface marker positive cells were determined by gating on the positive population in the appropriate histogram. (**b**) Proportions of CD3^+^CD4^+^CD44^hi^CD62L^low^LPAM-1^+^ cells (effector memory Th cells) in blood, spleen, and mLN at the indicated time points after MN vaccination. (**c**) Proportions of CD3^+^CD4^+^CD44^hi^CD62L^low^LPAM-1^+^ cells in blood, spleen, and mLN at the indicated time points after IM vaccination. Shown in (**a**) are representative data from a mouse on day 44, vaccinated with 5 µg IRV. Shown in (**b**) and (**c**) are the mean and s.d. of data from 4 or 5 mice. Mann-Whitney test was used for statistical analysis. Upper p value indicator for 5 µg IRV vaccination; lower p value indicator for 1 µg IRV vaccination. If only one p value indicator is present, this indicator applies to both, 1 µg and 5 µg IRV vaccination. ns: not significant p > 0.05; *p ≤ 0.05; **p ≤ 0.01; MN: microneedle; IM: intramuscular; mLN: mesenteric lymph node.

Immune cells expressing the gut-homing receptor LPAM-1 have an important role in clearing infections in the intestine and intestine-associated mucosa^24,25^. To analyze if IRV vaccination led to the expression of LPAM-1 on immune cells, we analyzed B cells, Th cells, and Tc cells two days after each vaccination in blood, spleen and mLN of mice (Figs 2 and 3; Supplementary Fig. 4). Since LPAM-1 positive cells are only present in a very low percentage in naïve animals, gating strategy is shown from a representative animal receiving 5 µg IRV delivered by MN patch (Figs 2a and 3a).

We found that LPAM-1 expression on B cells of vaccinated mice depended, to a certain degree, on dosing and vaccination route (Fig. 2). LPAM-1 expression on B cells in peripheral blood was significantly elevated (p = 0.0286 for 1 µg and 5 µg IRV) compared with MN placebo controls as early as two days after the first MN patch vaccination and then declined to baseline levels (Fig. 2b). The second and third vaccinations did not boost the expression of LPAM-1 on B cells in the peripheral blood. A significant increase in the expression of LPAM-1 on B cells appeared at day 2, post dose 1, in spleen (p = 0.0175 for 5 µg) and in mLN (p = 0.0159 for 1 µg and 5 µg) after MN patch vaccination, this expression further increased post dose 2 in spleen (p = 0.0313 for 1 µg and 5 µg) and in mLN (p = 0.0161 for 1 µg and p = 0.0038 for 5 µg) and post dose 3 in spleen (p = 0.0079 for 1 µg and 5 µg) and in mLN (p = 0.0119 for 1 µg and p = 0.0159 for 5 µg). On day 63, the proportions of LPAM-1 positive B cells dropped to 11% in mLN of mice that received 1 µg and 5 µg doses by MN patch, and 7%–8% in spleen of 1 µg and 5 µg IM vaccinated animals, respectively. Similar patterns were observed in IM vaccinated animals (Fig. 2c). However, we only saw a significant increase in the proportion of LPAM-1 positive B cells in mLN from post dose 2 (day 23) to study end (day 63), mostly in mice receiving 5 µg IRV (p = 0.0159, p = 0.0161, and p = 0.0119 for day 23, day 44, and day 63 respectively). In spleen, we only observed at study end (day 63) a significant elevation of LPAM-1 positive B cells in mice receiving high (p = 0.0119) and low (p = 0.0313) dose IRV via the IM route. Administration of 1 µg or 5 µg IRV by both vaccination routes induced a comparable increase in the proportions of activated, LPAM-1-expressing B cells in blood and immune organs of mice, resulting in significantly elevated LPAM-1-expressing B cell levels in spleen (p = 0.0159 for IRV MN; p = 0.0286 for IRV IM) and mLN (p = 0.0079 for IRV MN; p = 0.0159 for IRV IM) at study end (day 63). Thus, we demonstrated a potential dose sparing effect for parenterally administered IRV.

We observed similar patterns for effector memory Th cells expressing LPAM-1 (Fig. 3). Both vaccination routes at low and high doses induced similar increase of those cells in blood on day 2 (post dose 1), which declined to baseline levels during study period. In spleen and mLN of MN patch-vaccinated mice, we observed a significant increase in LPAM-1-expressing effector memory Th cells from day 23 (post dose 2; p = 0.0119 for 1 µg and 5 µg in spleen; p = 0.0286 for 1 µg and p = 0.0159 for 5 µg in mLN) through day 44 (post dose 3; p = 0.0119 for 1 µg and p = 0.0079 for 5 µg in spleen; p = 0.0159 for 1 µg and p = 0.0079 for 5 µg in mLN) (Fig. 3b). Comparable percentages of positive cells were detected in spleen of IM vaccinated mice, whereas substantial smaller percentage of positive cells were detected in mLN of IM vaccinated mice (Fig. 3c). At study endpoint (day 63), both vaccination routes led to significantly increased LPAM-1-expressing effector memory Th cells in spleen (p = 0.0159 for 1 µg and 5 µg IM; p = 0.0119 for 1 µg and 5 µg MN) and mLN (p = 0.0117 for 1 µg and p = 0.0114 for 5 µg IM; p = 0.0119 for 1 µg and 5 µg MN) compared to placebo controls. No differences in LPAM-1 expressing effector memory Th cells were observed in spleen and mLN between IM and MN patch vaccinations.

In addition, both MN patch and IM vaccinations induced elevated expression of LPAM-1 positive effector memory Tc cells in blood on day 2, which then declined during the study period (Supplementary Fig. 4). LPAM-1 expression on effector memory Tc cells showed only slight increase in spleen and mLN after MN patch or IM vaccination over the time course of this study. At study end (day 63), only high and low dose MN patch vaccinated mice showed a significant increase in LPAM-1-expressing effector memory Tc cells in spleen (p = 0.0119 for 1 µg and 5 µg) and mLN (p = 0.0498 for 1 µg and p = 0.0159 for 5 µg) compared to placebo controls. No significant increase could be shown for IM vaccination routes in blood, spleen, and mLN as well as blood from MN patch vaccinated mice.

### Differences in Th1 and Th2 cytokine induction after MN patch and IM vaccination

To determine if the induction of effector memory Th cells by IRV vaccination was associated with the presence of pro- and anti-inflammatory cytokines, peripheral blood of vaccinated animals was examined for 7 cytokines (Table 1). MN patch vaccination with 5 µg IRV induced elevated levels of IFN-γ on day 2 (post dose 1) but no other cytokines, indicative of a Th1-leaning response. IM vaccination with 5 µg IRV induced an increase of IL-6 on day 23 (post dose 2) and IL-4, IL-6, IL-10, and IFN-γ on day 44 (post dose 3), suggesting a balanced Th1/Th2 response. In addition, IM vaccination induced IL-17A, a Th17 associated cytokine on day 44 (post dose 3). Type I IFN response (IFN-α) was measured to determine the systemic activation of the innate immune system; we only observed a slight increase in IFN-α expression on day 44 (MN patch). No increase in TNF-α expression could be observed in any vaccination routes (Table 1). No increase of any cytokines analyzed could be observed after vaccination with 1 µg IRV independent of the vaccination route (Supplementary Table 1). These data indicate that MN patch vaccination induces a slight Th1 cytokine profile, whereas IM vaccination induces both Th1 and Th2 cytokine profiles in mice.

**Table 1:** Cytokine profiles in sera of mice that received IRV using a MN patch or by IM injection.

Cytokine	MN patch 5 µg				IM 5 µg			
	Baseline [pg/ml]	Day 2 [pg/ml]	Day 23 [pg/ml]	Day 44 [pg/ml]	Baseline [pg/ml]	Day 2 [pg/ml]	Day 23 [pg/ml]	Day 44 [pg/ml]
**Type I IFN**								
IFN-α	31.3 ± 3.4	29.9 ± 2.2	27.87 ± 7.5	134.9 ± 6.8	30.6 ± 3.9	34 ± 4.6	24.7 ± 7.6	33.64 ± 3.4
**Th1 cytokines**								
IFN-γ	31.3 ± 11.3	180.05 ± 4.46	81.26 ± 6.99	84.95 ± 3.34	33.23 ± 9.4	16.15 ± 13.3	29.03 ± 2.76	97.91 ± 42.13
IL-2	24.99 ± 5.42	58.71 ± 30.68	21.03 ± 2.53	21.8 ± 1.59	21.89 ± 2.3	13.06 ± 10.8	30.43 ± 18.96	22.1 ± 2.07
TNF-α	69.5 ± 13.1	87.03 ± 11.9	80.6 ± 13.9	75.7 ± 17.6	60.1 ± 3.1	60.8 ± 7.9	79.2 ± 22.3	63.4 ± 3.04
**Th2 cytokines**								
IL-4	33.7 ± 2.33	39.8 ± 6.43	33.65 ± 3	36.3 ± 4.13	39.3 ± 6.4	20.6 ± 17.1	33.42 ± 4.15	147.3 ± 11.4
IL-6	57.15 ± 9.2	69.54 ± 7	59.8 ± 9.6	78.9 ± 27.2	51.5 ± 1.4	35.4 ± 29.43	457.3 ± 23.42	254.2 ± 9.1
IL-10	228.9 ± 129.7	161.6 ± 40.14	137.03 ± 52.6	143.2 ± 41.8	158.42 ± 1.5	68.3 ± 55.9	162.4 ± 74.15	1197 ± 111.9
**Th17 cytokines**								
IL-17A	40.4 ± 3.75	53.4 ± 16.5	44.9 ± 2.8	45.6 ± 3.4	43.3 ± 2.8	25.1 ± 19.7	47.6 ± 6.5	191.5 ± 14

Mice were vaccinated with 5 µg IRV via microneedle (MN) patch or via intramuscular (IM) injection. Serum samples collected at the indicated time points were analyzed for the presence of Th1, Th2, and Th17 cytokines by a flow cytometry-based bead array or by IFN-α ELISA as described in the text. Data shown are the mean and s.d. from 4 (baseline) or 5 mice. Cytokine concentrations are shown in [pg/ml].

### IRV by MN patch and IM vaccination induces comparable levels of IgG and neutralizing antibodies

**Figure 4:**
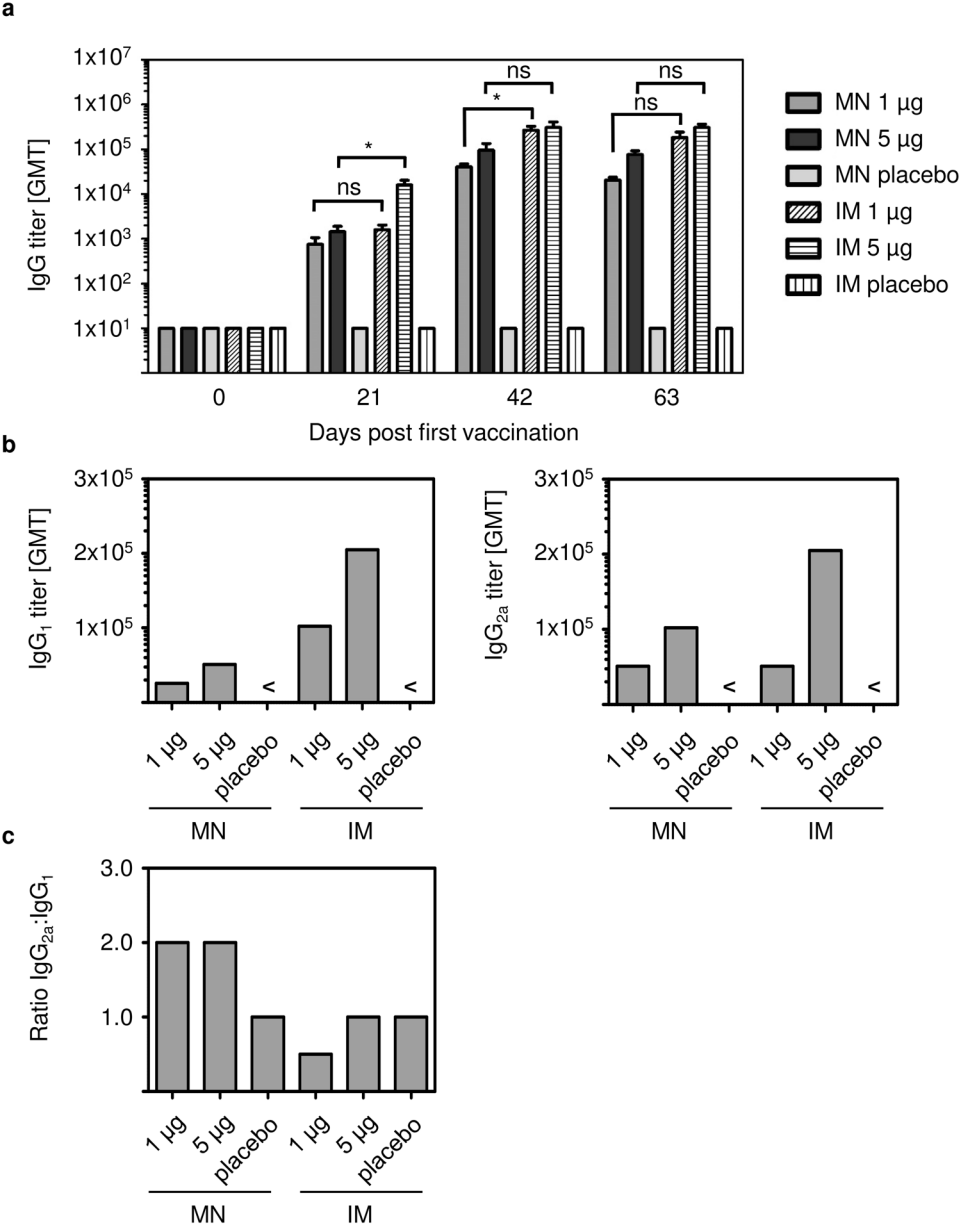
IRV vaccination leads to elevated levels of rotavirus-specific IgG and IgG subclass in sera of mice. Peripheral blood was collected before the mice received the first vaccination dose (day 0), before the second and third vaccination dose (day 21, day 42) and at the study endpoint (day 63). (**a**) Individual serum samples or (**b**) pooled serum samples from 5 mice were tested for IgG and IgG_1_ and IgG_2a_, respectively, by ELISA as described in the text. (**c**) IgG_2a_:IgG_1_ ratio was calculated from antibody levels detected in (**b**). Shown in (**a**) are mean and s.d. of n = 5. Mann-Whitney test was used for statistical analysis. MN: microneedle; IM: intramuscular; < : below detection limit; ns: not significant; *p ≤ 0.05; **p ≤ 0.01.

We analyzed the levels of RV-specific IgG antibodies in sera of IM and MN patch-vaccinated mice collected on days 0, 21, 42, and 63 (Fig. 4a). RV-specific IgG was detected after one dose vaccination, and its levels increased after two vaccinations and remained elevated up to 63 days. Placebo vaccinated mice had no elevated IgG levels throughout the study. Of note, low dose and high dose IRV induced comparable IgG levels on days 42, and 63 for IM vaccination. MN 1 µg IRV induced slightly lower levels of total IgG antibodies compared to MN 5 µg IRV over the time course of this study, but those differences were not statistically significant. In addition, no significant differences were seen between IM and MN vaccination for both doses at study end (p = 0.0503 for 1 µg; p = 0.0527 for 5 µg). These data indicate a dose-sparing effect independent on the vaccination route.

We further analyzed sera for IgG subclasses, IgG_1_ and IgG_2a_ (Fig. 4b). Both vaccination routes induced IgG_1_ and IgG_2a_ in a dose-dependent manner. Overall, IM vaccination induced higher levels of IgG_1_ and IgG_2a_ antibodies than MN patch vaccination. However, vaccination with 1 µg IRV by MN patch and IM injection led to comparable IgG_2a_ titers at study end. MN patch vaccination at both doses resulted in a higher IgG_2a_:IgG_1_ ratio than IM vaccination (Fig. 4c). Overall, these data show that MN patch vaccination with IRV induced a slightly more dominant Th1 response, whereas IM vaccination led to a balanced Th1/Th2 response. Of note, no elevated IgA levels compared to baseline control were detected in serum after MN patch and IM vaccination (data not shown).

**Figure 5:**
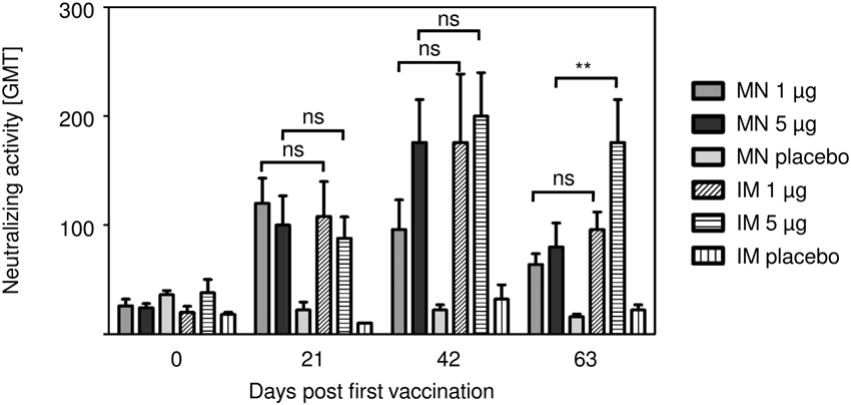
IRV vaccination induces elevated levels of rotavirus-specific neutralizing activity in mice. Peripheral blood was collected before the mice received the first (day 0), the second (day 21) and third (day 42) vaccination dose, and at the study endpoint (day 63). Levels of rotavirus-specific neutralizing activity in serum was determined by a microneutralization assay as described in the text. Data show mean and s.d. of n = 5. Mann-Whitney test was used for statistical analysis. MN: microneedle; IM: intramuscular; ns: not significant; **p ≤ 0.01.

Last, we assessed the neutralizing activities in sera of IRV-vaccinated mice (Fig. 5). Mice that received two doses of 1 µg and 5 µg IRV by MN patch or IM injection had comparable levels of neutralizing activities against the homotypic RV strain Wa. For vaccination with the MN patch delivering 1 µg, no boosting effect was observed after two additional vaccinations (days 42 and 63). Of note, on day 63 neutralizing activity titer for groups receiving 1 µg dose by both MN patch and IM vaccination declined, but remained significantly above the levels of the appropriate placebo controls. Only in IM 5 µg vaccinated animals, Wa-neutralizing antibody titers remained significantly elevated (p = 0.0277).

## Discussion

The present study is the first to perform a comprehensive evaluation of systemic and mucosal immunity to an IRV by IM injection and dissolvable MN patch application. IRV vaccination using MN patch and IM injection induced LPAM-1-expressing Th and B cells in spleen and mLN of vaccinated mice. Expression of LPAM-1 especially in mLN is crucial to protect the vaccinated individual from infection with gastrointestinal viruses^26,27^. While MN patch vaccination induced a comparable level of LPAM-1-expressing B cells and Th cells in spleen and mLN, IM vaccination led to a higher percentage of LPAM-1 positive B cells in mLN and more Th cells in the spleen. This is in accordance with published data that generally showed the recruitment of LPAM-1 positive memory T cells to mucosa-associated lymphoid organs^20^. We analyzed LPAM-1 positive effector memory Th cells, which are known for their potential to migrate especially to peripheral tissues including intestinal mucosa, skin or lungs^28^ and can secrete effector cytokines like IFN-γ and IL-4^29^. We observed LPAM-1 induction on B and Th cells in the blood as early as 2 days post vaccination but we did not observe a boosting effect for this cell population in the blood. Most leucocytes pass through the blood stream only once during their migration^30^, which may explain the phenomenon observed in this study. LPAM-1 expression on B and Th cell is induced potentially in draining LN after contact with IRV followed by a migration towards the intestinal mucosa and the spleen. Future studies would need to examine if LPAM-1-expressing B and Th cells are migrating through the blood to mLN and spleen at other time points after IRV vaccination.

MN patches facilitate vaccination via the skin, which has the advantage of directly targeting APC and triggering a strong immune response with a low antigen dose^14,31,32^. In the present study, MN patch- and IM-administered IRV at low and high dose induced comparable humoral and cellular immune responses as well as similar induction of LPAM-1-expressing B and effector memory Th cells. Our observations of dose-sparing potential hold promise for a low cost IRV for use in global immunization.

MN patch vaccination appeared to induce higher production of IFN-γ and RV-specific IgG_2a_, thus a Th1 immune response, whereas IM vaccination induced comparable Th1 and Th2 cytokines, and similar levels of IgG_2a_ and IgG_1_ antibodies, indicating a balanced Th1 and Th2 response. Our findings are in agreement with those in mice that received RV VLPs by IM injection or IRV using a coated stainless steel MN patch^14,33^. MN patch vaccination in mice also led to a strong induction of Th1 response to an H1N1 flu vaccine and production of fewer pro-inflammatory cytokines than IM vaccination^34,35^. Th cell responses differ according to the antigen uptake and the nature of stimulating agent. Thus, it is likely that IM and MN patch administrations used here contributed to different uptake by APC and could explain the differences in Th1 and Th2 response.

We observed some reduction in neutralizing activity in MN patch-vaccinated mice at study end despite comparable titers of total IgG in IM and MN patch vaccinated animals. The reasons for this drop in neutralizing activity are not known and may reflect a property of RV vaccination in skin. However, studies with other vaccines have shown long duration of immunity after MN patch vaccination compared to IM delivery^36–38^. In addition, MN patch vaccination in this study was performed without an adjuvant, whereas IM vaccination was adjuvanted with alum, which has been shown to enhance and shape innate and adaptive immunity^39^. Of note, our early studies showed that second and third dose of CDC-9 IRV when formulated with alum and administered IM further boosted neutralizing activity to homotypic and heterotypic human rotavirus strains in guinea pigs and gnotobiotic piglets^18,19,40^.

In conclusion, we demonstrated that CDC-9 IRV by MN patch vaccination without an adjuvant or IM administration with alum-adjuvantation induced RV-specific systemic and intestinal immune responses, including the induction of effector memory cells expressing the gut homing receptor LPAM-1 in mice. MN patch vaccination produced more Th1 phenotype, whereas IM vaccination induced a more balanced Th1/Th2-phenotype, as indicated by cytokine and IgG subclass induction. The findings of LPAM-1-expressing B and Th cells in blood, spleen and mLN of IRV-vaccinated mice could help us investigate and define correlates of protection from parenteral RV vaccination in animals and humans. IRV by IM administration or by MN patch is a promising alternative and improvement to oral RV vaccination among children throughout the world.

## Methods

### Vaccine preparation

CDC-9, a human G1P^8^, RV strain was cultivated in Vero cells^14^. Triple-layered particles (TLPs) were purified from cell culture supernatants by using isopycnic CsCl gradient centrifugation and inactivated at 60 °C for 4 hours^2^. TLPs in HBSS supplemented with 10% sorbitol were mixed with 2.96% w/w aluminum hydroxide (Alhydrogel, Accurate Chemical & Scientific Corp, Westbury NY) on a rocking platform overnight at 4 °C. The vaccine was stored at 4 °C before use for intramuscular (IM) injection.

Inactivated TLPs were used for the fabrication of dissolving polymer MN patches^41^. The fabrication was a two-step molding process on PDMS molds. Patches contained 100 conical MN in a 10 × 10 pattern (850 µm height and 500 µm diameter at base). HBSS was used to perform all necessary dilutions. IRV was formulated at a concentration of 0.4 or 2 mg/ml IRV with 1% (w/v) sodium carboxymethylcellulose, and 5% (w/v) sorbitol, and cast on PDMS molds. Vacuum was applied to ensure filling of the solution into the mold. Excess solution was removed after 40 minutes from the mold and the filled mold was left on vacuum for 20 additional minutes. The second filling solution, containing 18% PVA and 18% sucrose, was cast on the PDMS mold under vacuum for 3 hours and dried overnight at 35 °C. Placebo MN patches, containing no IRV, were prepared in the same manner. MN patches were produced two days before vaccination of animals and stored at room temperature. Delivery efficiency was expected to be ~50%, therefore patches were designed to contain 2 µg IRV and 10 µg IRV for the delivery of 1 µg and 5 µg, respectively. Four patches were analyzed for antigen concentration before application into mice with Premier Rotaclone EIA (Meridian Bioscience, Cincinnati, OH).

### Vaccination of mice

Balb/c mice (8–10 weeks old) were purchased from Charles River Laboratories (Wilmington, MA). Mice were anesthetized with intraperitoneal injection of 110 mg/kg ketamine (Vet One, Boise, ID) and 11 mg/kg xylazine (Akorn Animal Health, Lake Forest, IL). For MN patch application, the back of mice was shaved with an electric shear and remaining hair was removed using a depilatory cream (Nair, Church & Dwight, Princeton, NJ) two days before patch application. Pre-bleed was taken from the submandibular vein. Serum and organs were collected from 4 unvaccinated mice for baseline assessment. Mice in groups of 20 were anesthetized before each vaccination and vaccinated with 1 µg or 5 µg IRV formulated with alum or alum only (placebo control) by IM injection into the upper quadriceps muscle, or with MN patch containing 2 µg or 10 µg IRV MN patch or placebo patch, which was applied with pressure for 30 seconds on the back of the animals. After 15 minutes on the skin, MN patches were removed, analyzed microscopically for structural integrity, and then reconstituted in 0.5 ml HBSS to determine the residual antigen amount by Premier Rotaclone EIA (Meridian Bioscience, Cincinnati, OH). Measured antigen concentration of 4 patches and the determined residual antigen amount were used to calculate the delivered dose. Five mice from each group were sacrificed 2 days after each vaccination to examine changes in serum cytokine profiles, immune cell distribution, and gut-homing marker expression. All experiments were approved by the Institutional Animal Care and Use Committee (IACUC) of the CDC and conducted in accordance with the ethical guidelines for animal experiments and safety guidelines.

### Quantification of cellular immune responses by flow cytometry

Blood was collected at the indicated time points by submandibular vein puncture and sera were prepared. Murine cytokines in sera were measured by using the BD Cytometric Bead Array for Murine Th1/Th2/Th17 cytokines (BD Bioscience, San Jose, CA) according to manufacturer’s instructions. Murine IFN-α in sera was detected by using VeriKine Mouse IFN Alpha ELISA Kit (PBL Assay Science, Piscataway, NJ) according to manufacturer’s instructions.

### Cell isolation

Following euthanasia of mice with CO_2_, spleen and mesenteric lymph nodes (mLN) were harvested. Splenocytes were isolated by dissecting the spleen capsule and pressing with a pipette. Cells were filtered through a 70 µm cell strainer and centrifuged at 1200 rpm for 5 minutes followed by red blood cell lysis (Red Blood Cell Lysing Buffer Hybri-Max, Sigma-Aldrich, St. Louis, MO). Single cell suspension from mLN was prepared by mashing mLN through a 70 µm cell strainer followed by centrifugation at 1200 rpm for 5 minutes.

### Flow cytometric analysis

Single cell suspensions isolated from spleen and mLN, and whole blood containing peripheral blood mononuclear cells (PBMC) were incubated with cell surface marker-specific fluorescence-labelled antibodies for 20 minutes at 4 °C. Antibodies targeting the following cell surface markers were used: anti-CD11c-FITC (clone N418), anti-NK1.1-PE (clone PK136), anti-CD86-PE/Cy7 (clone GL-1), anti-CD4-Pacific Blue (clone RM4–5), anti-CD11b-Pacific Blue (clone M1/70), anti-CD45R/B220-APC (clone RA3-6B2), anti-CD8-Alexa Fluor 700 (clone 53–6.7), anti-CD80-Pacific blue (clone 16–10A1), anti-CD44-Pacific Blue (clone IM7), anti-CD62L-APC (clone MEL-14), anti-LPAM-1-PE (clone DATK32), anti-Gr-1-APC (clone RB6-8C5) (all BioLegend, San Diego, CA); anti-CD3-FITC (clone C363.29B, Southern Biotech, Birmingham, AL); and anti-F4/80-PE (clone Cl:A3-1, Bio-Rad, Raleigh, NC). After staining of whole blood PBMC, BD FACS lysing solution (BD Bioscience, San Jose, CA) was added for red blood cell (RBC) lysis and fixation of the cells. Stained single cell suspension from spleen, mLN and RBC-lysed blood were washed once and fixed with 1% PFA. Cells were analyzed using BD LSRFortessa with BD FACS Diva software (BD Bioscience, San Jose, CA) and FlowJo software (FlowJo, LLC, Ashland, OR).

### Quantification of antibodies by ELISA and microneutralization assay

RV-specific IgG, IgA, and IgG subclass antibodies were detected in sera on day 0, 21, 42, and 63 as described previously^2,19,33^. In brief, 96-well plates were coated with rabbit hyperimmune serum to RV Wa overnight at 4 °C. The plates were washed, blocked with 5% skim milk in PBS followed by an incubation with supernatants of rhesus RV (RRV)-infected MA104 cells (~10^6^ FFU/ml) for 1 hour at 37 °C. Serial diluted mouse serum samples were added and incubated for 2 hours at 37 °C. Detection occurred with HRP-conjugated goat anti-mouse IgG, HRP-conjugated goat anti-mouse IgA, HRP-conjugated goat anti-mouse IgG_1_ or HRP-conjugated goat anti-mouse IgG_2a_ (all Southern Biotech, Birmingham, AL). TMB (Sigma Aldrich, St. Louis, MO) substrate was added for color development and reaction was stopped with 1 N HCl. Optical density (OD) was determined with an EIA reader (Dynex Technologies, Chantilly, VA). The antibody titer in the serum was defined as the reciprocal of the highest dilution giving a mean OD greater than the cutoff value of 0.1. Each serum specimen was tested at an initial dilution of 1:10 for IgA and 1:100 for IgG. If IgA or IgG activity was not detected at initial dilution, a value of 1 for IgA and 10 for IgG was used for the calculation of geometric mean titer (GMT).

RV-specific neutralizing activity was measured in a microneutralization assay against RV strain Wa^42^. In brief, mouse sera from days 0, 21, 42, and 63 were diluted two-fold in duplicates in 96-well plates and incubated with 600 FFU of Wa per well for 1 hour at 37 °C. MA104 cells in IMDM with 10 µg/µl trypsin (Invitrogen, Carlsbad, CA) and 0.5% chicken serum (Invitrogen, Carlsbad, CA) were added to each well and incubated at 37 °C for 3 days in a humidified incubator. The plates were fixed with formalin and incubated with rabbit hyperimmune serum. RV antigen was detected with HRP-labeled goat anti-rabbit antibody and color was developed with TMB. Neutralizing antibody titer was defined as the reciprocal of the highest dilution that gave a greater than 70% reduction in the absorbance value when compared to virus-only controls^14^.

### Statistical analysis

Graphs and statistical analysis were performed using GraphPad Prism 5.02. To evaluate the differences in induction of LPAM-1 expression on immune cells (unpaired data), IgG antibody induction, and induction of neutralizing activity, we assumed no normal distribution of data and used the Mann-Whitney test (two tailed). P values lower than 0.05 were considered as statistically significant with p ≤ 0.05 (*) and p ≤ 0.01 (**).

### Data availability

The data generated and analyzed during this study are available with the article and its Supplementary Information files, or are available from the corresponding author upon reasonable request.

The findings and conclusions in this report are those of the authors and do not necessarily represent the official position of the Centers for Disease Control and Prevention.

## Electronic supplementary material

Supplementary Material
