# Supplementary material for: Inactivated rotavirus vaccine by parenteral administration induces mucosal immunity in mice
Source: Sci Rep. 2018 Jan 12;8:561. doi: 10.1038/s41598-017-18973-9 (PMC5766576; doi:10.1038/s41598-017-18973-9)
Supplement: Supplementary file 1 — Supplementary Material [file 41598_2017_18973_MOESM1_ESM.pdf]

Inactivated rotavirus vaccine by parenteral administration induces mucosal immunity in mice

Theresa K Resch[1], Yuhuan Wang[1], Sung-Sil Moon[1], Jessica Joyce[2], Song Li[2], Mark Prausnitz[2], and Baoming Jiang[1*]

[1]Division of Viral Diseases, Centers for Disease Control and Prevention (CDC), Atlanta, Georgia, USA
[2]School of Chemical & Biomolecular Engineering, Georgia Institute of Technology, Atlanta, Georgia, USA

*Address of correspondence: Dr. Baoming Jiang, Viral Gastroenteritis Branch, National Center for Immunization and Respiratory Diseases, MS G04, 1600 Clifton Road NE, Atlanta, GA 30329. Fax: 404-639-3645. E-mail: bxj4@cdc.gov.

The findings and conclusions in this report are those of the authors and do not necessarily represent the official position of the Centers for Disease Control and Prevention.

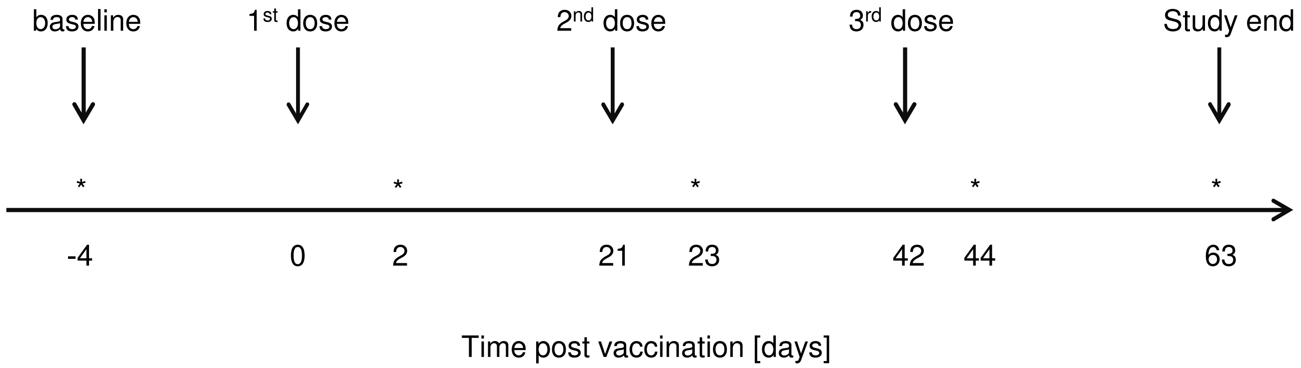

Supplementary figure 1. Schematic view of the vaccination schedule. 4 Animals were analyzed for baseline cytokine expression and immune cell distribution 4 days before vaccination. Organs of animals were harvested and analyzed by flow cytometry. On day 0 mice were vaccinated with IRV delivered either intramuscular (IM) or with the microneedle (MN) patch. Delivered doses were 1 µg and 5 µg for each vaccination route. Second dose was applied on day 21, third dose at day 42. Mice were bleed directly before each vaccination. After each vaccination dose, blood was withdrawn via the submandibular vein at 24 hours and 48 hours. Asterisks indicate harvest of organs from 5 mice of each group (*) for flow cytometric analysis of immune cell distribution (two days after each vaccination). Study end was on day 63 after the first vaccination.

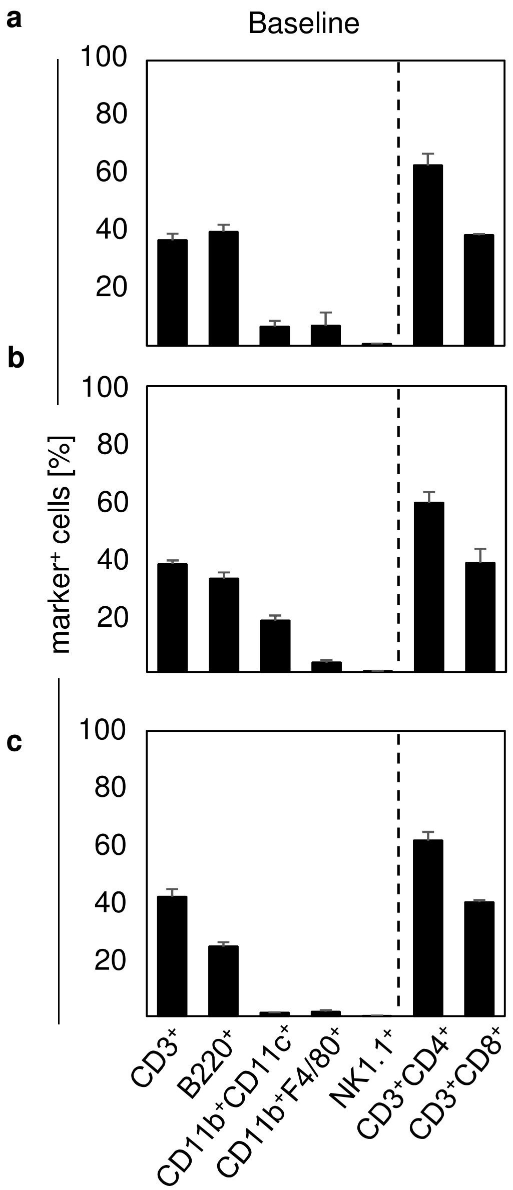

a. Spleen
b. Blood
c. mLN

Supplementary Figure 2. Immune cell distribution in spleen, peripheral blood and mesenteric lymph nodes (mLN) of baseline animals. Blood **(a)**, spleen **(b)**, and mLN **(c)** were collected from unvaccinated mice (baseline). Immune cells were stained with fluorescence-labelled antibodies directed against the indicated surface molecules. All flow cytometric analysis throughout this study were performed by gating on living cells in the FSC/SSC plot. Percentage of surface marker positive cells were determined by gating on the positive population in the appropriate histogram. Percentage of $CD3^+CD4^+$ and $CD3^+CD8^+$ were calculated from total $CD3^+$ cells. Data shown is median and s.d. of n=5. $CD3^+$ (T cells), $B220^+$ (B cells), $CD11b^+CD11c^+$ (Granulocytes), $CD11b^+F4/80^+$ (myeloid cells), $NK1.1^+$ (NK cells), $CD3^+CD4^+$ (T helper cells), $CD3^+CD8^+$ (cytotoxic T cells). mLN: mesenteric lymph node.

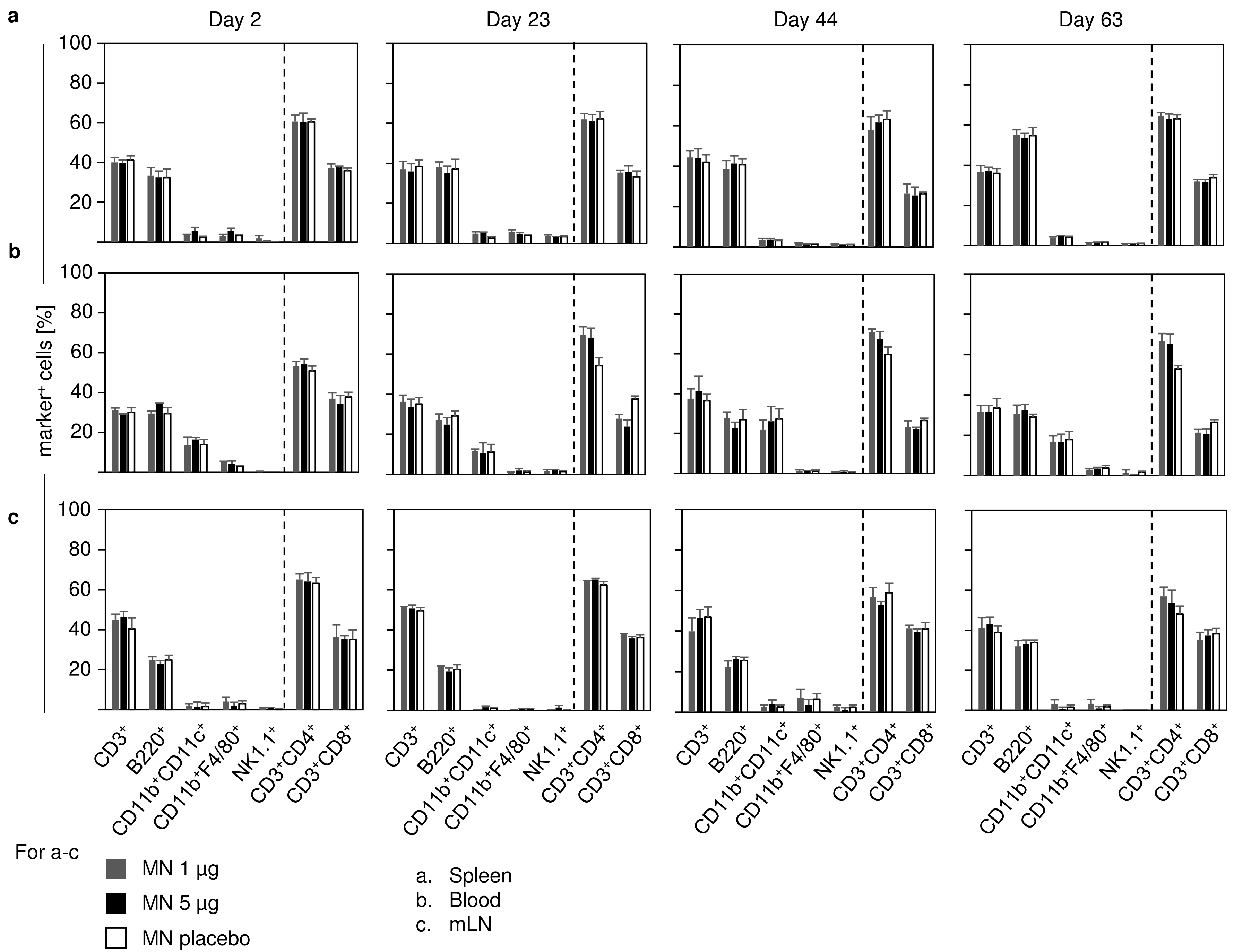

a
b
c
Day 2
Day 23
Day 44
Day 63
100
80
60
40
20
marker⁺ cells [%]
CD3⁺
B220⁺
CD11b⁺CD11c⁺
CD11b⁺F4/80⁺
NK1.1⁺
CD3⁺CD4⁺
CD3⁺CD8⁺
For a-c
MN 1 µg
MN 5 µg
MN placebo
a. Spleen
b. Blood
c. mLN

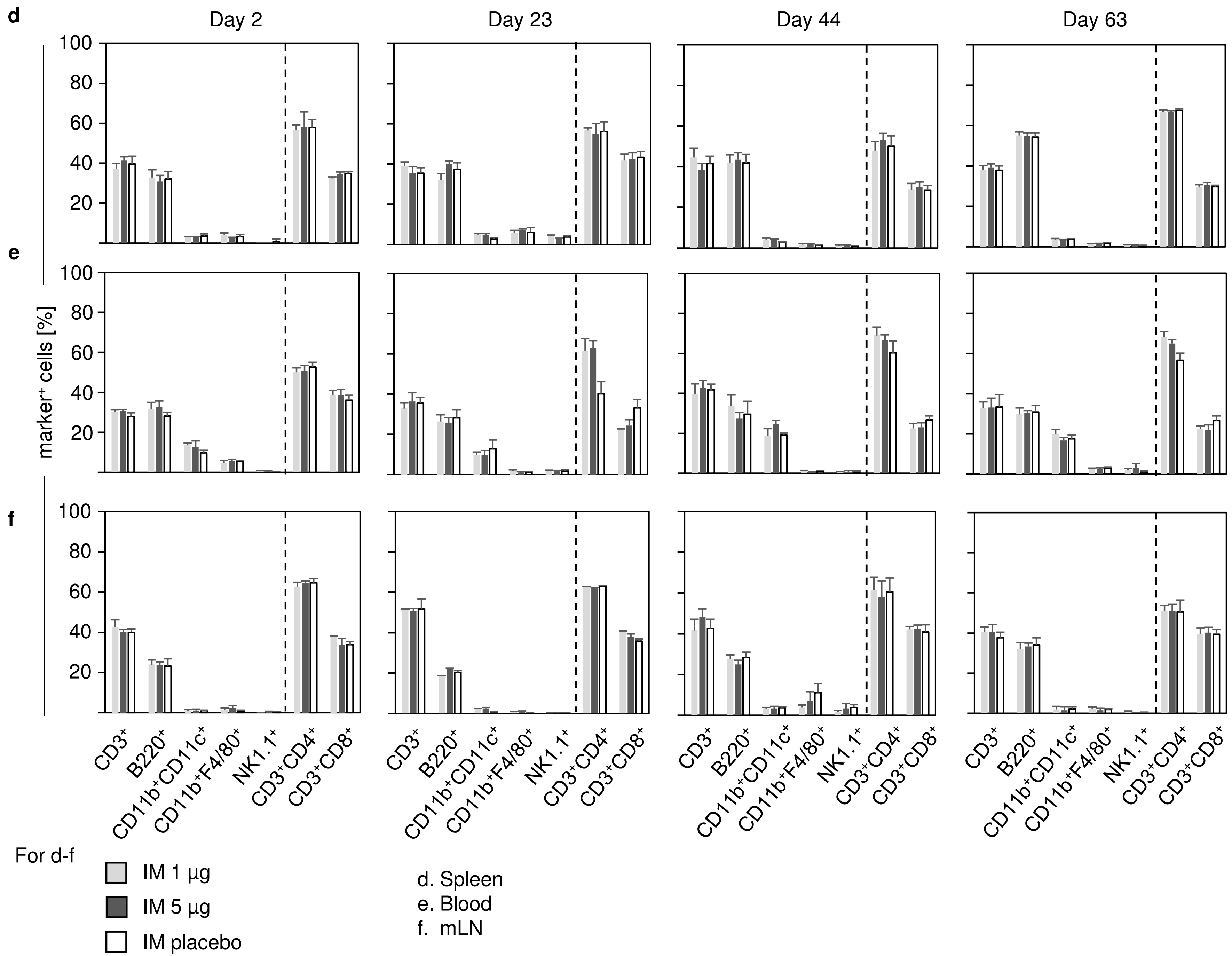

d
e
f
Day 2
Day 23
Day 44
Day 63
marker⁺ cells [%]
100
80
60
40
20
CD3⁺
B220⁺
CD11b⁺CD11c⁺
CD11b⁺F4/80⁺
NK1.1⁺
CD3⁺CD4⁺
CD3⁺CD8⁺
For d-f
IM 1 µg
IM 5 µg
IM placebo
d. Spleen
e. Blood
f. mLN

Supplementary Figure 3. IRV vaccination does not affect the overall immune cell distribution in spleen, peripheral blood and mesenteric lymph nodes (mLN). Mice were vaccinated with microneedle (MN) patch **(a-c)** or by intramuscular (IM) injection **(d-f)** delivering 1 µg or 5 µg IRV or the respective placebo controls. Analysis of immune cell distribution in spleen (a and d), peripheral blood (blood) (b and e), and mesenteric lymph nodes (mLN) (c and f) was done two days after each vaccination dose (day 2, 23 and 44) as well as at the study end point (day 63). Immune cells were stained with fluorescence-labelled antibodies directed against the indicated surface molecules. All flow cytometric analysis throughout this study were performed by gating on living cells in the FSC/SSC plot. Percentage of surface marker positive cells were determined by gating on the positive population in the appropriate histogram. Percentage of $CD3^+CD4^+$ and $CD3^+CD8^+$ were calculated from total $CD3^+$ cells. Data shown is median and s.d. of n=5. $CD3^+$ (T cells), $B220^+$ (B cells), $CD11b^+CD11c^+$ (Granulocytes), $CD11b^+F4/80^+$ (myeloid cells), $NK1.1^+$ (NK cells), $CD3^+CD4^+$ (T helper cells), $CD3^+CD8^+$ (cytotoxic T cells). mLN: mesenteric lymph node; MN: microneedle; IM: intramuscular.

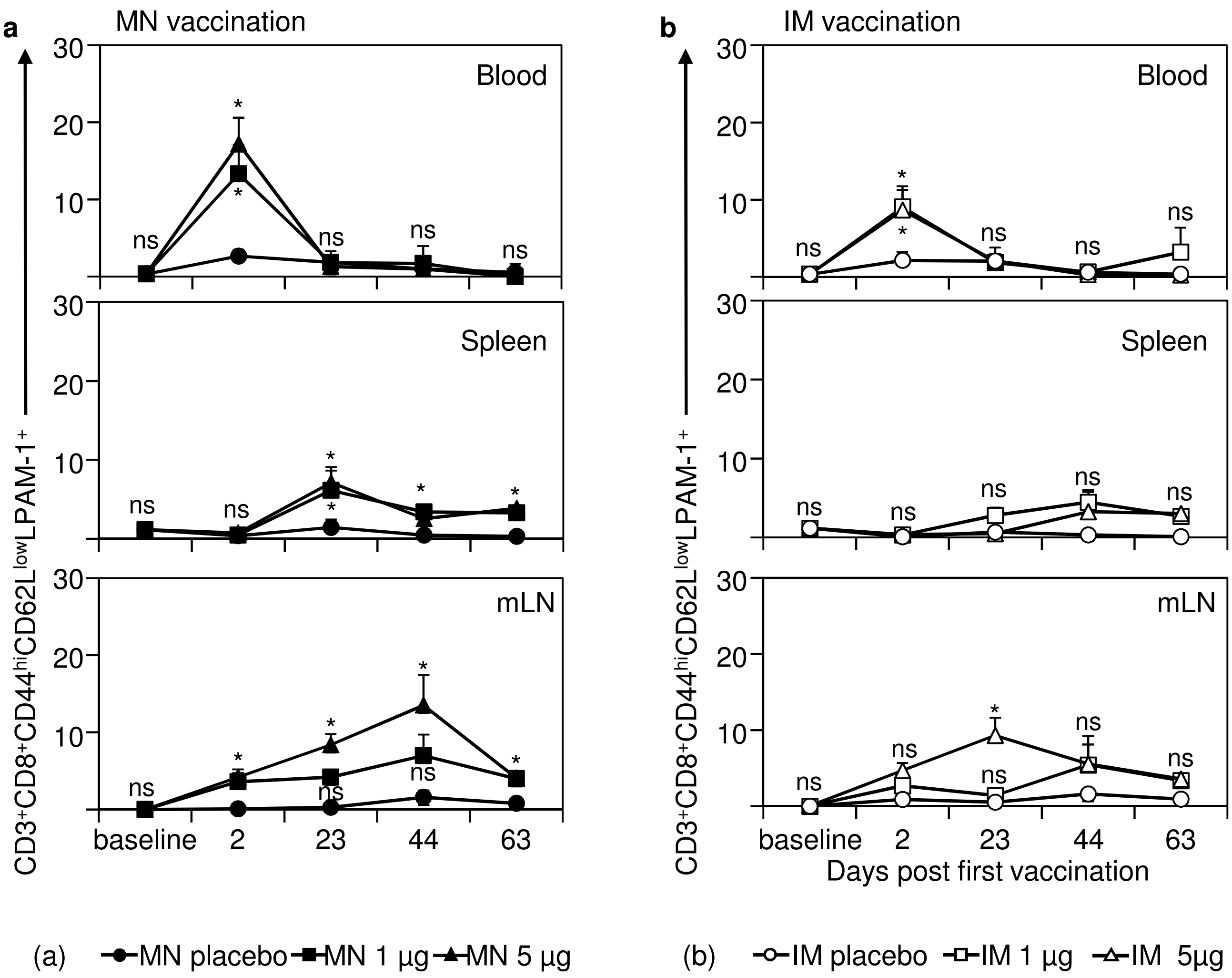

Supplementary Figure 4. Cytotoxic T cells expressing the gut homing receptor LPAM-1 are present early in the blood but absent in spleen and mesenteric lymph node (mLN) after IRV vaccination. Mice were vaccinated with IRV either via the microneedle (MN) patch **(a)** or intramuscular (IM) **(b)** in the indicated doses or with a placebo control. Organs were harvested and peripheral blood was collected 2 days after each dose (day 2, 23, and 44) and at the study end (day 63) compared to unvaccinated animals (baseline). Single cell suspension was stained with fluorescence-labelled antibodies directed against the indicated surface molecules. All flow cytometric analysis throughout this study were performed by gating on living cells in the FSC/SSC plot. Percentage of surface marker positive cells were determined by gating on the positive population in the appropriate histogram. Shown here are cytotoxic effector memory T cells expressing the gut homing receptor (CD3+CD8+CD44^{hi}CD62L^{low}LPAM-1^{+} cells). Data shown here are the mean and s.d. of n=5; baseline n=4. Mann-Whitney test was used for statistical analysis. Upper p value indicator for 5 µg IRV vaccination; lower p value indicator for 1 µg IRV vaccination. If only one p value indicator is present, this indicator applies to both, 1 µg and 5 µg IRV vaccination. ns: not significant $p > 0.05$; *: $p \leq 0.05$; **: $p \leq 0.01$; MN: microneedle patch; IM: intramuscular; mLN: mesenteric lymph node.

Supplementary Table 1: Cytokine profiles in sera of mice that received 1µg IRV using a MN patch or by IM injection or placebo.

Cytokine/dose		MN patch				IM			
		Baseline [pg/ml]	Day 2 [pg/ml]	Day 23 [pg/ml]	Day 44 [pg/ml]	Baseline [pg/ml]	Day 2 [pg/ml]	Day 23 [pg/ml]	Day 44 [pg/ml]
**Type I IFN**									
**IFN-α**	1 µg	33.3 ± 5.6	34.3 ± 5.7	36.3 ± 3.7	33.8 ± 4.6	30.6 ± 3.9	34 ± 4.6	24.7 ± 7.6	33.6 ± 3.4
	Placebo	26.3 ± 4.3	33.6 ± 4.6	34.8 ± 6.8	51.3 ± 11.1	30.6 ± 9.8	29.5 ± 2.8	34 ± 0.9	31.7 ± 3.6
**Th1 cytokines**									
**IFN-γ**	1 µg	28.4 ± 5.9	23.4 ± 1.4	35.5 ± 3.7	30.4 ± 3.2	30.1 ± 6.3	28.9 ± 1.7	37.1 ± 13.6	26.8 ± 1.1
	Placebo	29.9 ± 6.6	23.3 ± 8.5	39.9 ± 7.3	31.8 ± 8.1	32.6 ± 11.5	33.4 ± 11.4	31.4 ± 5.7	25.7 ± 1.9
**IL-2**	1 µg	20.9 ± 1.5	21.2 ± 2.8	27.9 ± 5.2	21 ± 0.9	23.5 ± 3.7	21.6 ± 1.7	32.5 ± 12.3	20.6 ± 1.7
	Placebo	19.2 ± 0.4	22.5 ± 2.2	24.3 ± 2.6	22.7 ± 4.4	26.9 ± 12.4	21.6 ± 1.7	21.9 ± 1.7	21.2 ±1.4
**TNF-α**	1 µg	64.9 ± 4.3	69.2 ± 7.3	66.8 ± 11.3	70.4 ± 6.6	73.8 ± 29.7	71 ± 5.7	72.2 ± 10.9	67.1 ± 4.8
	Placebo	61.5 ± 13.6	64.9 ± 12.8	74.2 ± 12.1	75.2 ± 12.4	62.4 ± 7.2	83.5 ± 20.6	86.1 ± 29.9	61.4 ± 2.4
**Th2 cytokines**									
**IL-4**	1 µg	33.9 ± 1.9	33.3 ± 4.4	32.5 ± 3.9	35.3 ± 3.7	44 ± 21.2	34.1 ± 5.5	33 ± 1.9	34.7 ± 1.8
	Placebo	30.8 ± 2.8	34.6 ± 6.4	40.9 ± 8.3	37.3 ± 9.2	33.2 ± 2.6	35.4 ± 6.1	36.5 ± 2	32.9 ± 2.1
**IL-6**	1 µg	53.6 ± 4.6	59.8 ± 9.6	54.7 ± 5.7	56 ± 1.6	50.1 ± 3.9	57.9 ± 4.2	76.6 ± 6	83.2 ± 17.9
	Placebo	54.6 ± 9.6	66.1 ± 8.3	69.7 ± 9.8	57.6 ± 2.9	50.9 ± 5.4	56.2 ± 5.5	66.7 ± 12.3	54.2 ± 6.7
**IL-10**	1 µg	117.7 ± 22.2	101.8 ± 9.2	119.1 ± 21.3	120.5 ± 20.5	171.6 ± 40	155.4 ± 16.9	137.7 ± 15.8	121.6 ± 19.4
	Placebo	128.6 ±35.3	85.4 ± 16.7	146.4 ± 25.6	106.5 ± 25.4	118.2 ± 17.2	117.9 ± 12.8	116.1 ± 13.3	97.2 ± 16
**Th17 cytokines**									
**IL-17A**	1 µg	42.1 ± 4.1	39.2 ± 1.3	46.5 ± 9.9	45.2 ± 4.9	41.8 ± 2.5	47.6 ± 9.5	48.8 ± 7.4	42.6 ± 3.9
	Placebo	42.3 ± 2.7	36.8 ± 10.1	52.7 ± 6.5	48.3 ± 9.1	42.5 ± 4.1	39.9 ± 2.4	43.3 ± 2.4	42.8 ± 4.7

Mice were vaccinated with 1 µg IRV or with the placebo control via microneedle (MN) patch or via intramuscular (IM) injection. Serum samples collected at the indicated time points were analyzed for the presence of Th1, Th2, and Th17 cytokines by a flow cytometry-based bead array or by IFN-α ELISA as described in the text. Data shown are mean and s.d. from n=4 (baseline) or n=5 mice. Cytokine concentrations are shown in [pg/ml].
